# Whey Versus Casein as a Protein Source during the Weaning Period: Impact on Growth and Later Adiposity and Glucose Homeostasis in a Rat Model of Intrauterine Growth Restriction

**DOI:** 10.3390/nu12113399

**Published:** 2020-11-05

**Authors:** Yasaman Shahkhalili, Florence Blancher-Budin, Cathriona Monnard, Julie Moulin, José Sanchez-Garcia, Katherine Macé

**Affiliations:** Société des Produits Nestlé S.A., 1000 Lausanne, Switzerland; yasishahkhalili@yahoo.fr (Y.S.); florence.blancher@gmail.com (F.B.-B.); julie.moulin@rdls.nestle.com (J.M.); Jose-Luis.SanchezGarcia@rd.nestle.com (J.S.-G.); catherine.mace@rdls.nestle.com (K.M.)

**Keywords:** whey, casein, weaning diet, glucose, insulin, IUGR

## Abstract

The impact of early life protein source (whey vs. casein) on short- and long-term glucose homeostasis and adiposity is unknown and was investigated in this study. At the end of the suckling period, non-IUGR (intrauterine growth restriction) and IUGR pups were separated from dams and were randomized into four groups. From age 21–49 days, non-IUGR and IUGR pups were fed ad-libitum chow or a semi-synthetic diet (20% from protein; casein or whey) and from age 50–199 days, all groups were fed ad-libitum chow. Food intake, body composition, glucose, and insulin homeostasis were assessed. Among the chow groups, IUGR had slower growth and higher fasting glucose at age 42 days, as well as higher fasting and AUC glucose at age 192 days relative to non-IUGR. The whey IUGR group had a slower growth rate and higher fasting glycemia in early life (age 21–49 days) and higher HOMA-IR later in life (age 120–122 and 190–192 days) relative to casein IUGR. This study shows the potential advantage of casein relative to whey during weaning on short term energy intake, growth, and glucose homeostasis in an IUGR model and reveals, for the first time, its long term impact on insulin sensitivity, which may have implications for later metabolic health, particularly in small-for-gestational-age populations at risk of type 2 diabetes.

## 1. Introduction

Intrauterine growth restriction (IUGR) results in reduced birth weight and subsequent rapid catch up growth, which are considered as independent risk factors for later development of chronic non-communicable, metabolic diseases. Barker et al. have proposed that suboptimal environmental factors, which hinder growth in utero, lead to long lasting alterations in the structure and function of developing tissues, as well as changes in the neuroendocrine system [1,2]. According to this hypothesis, such fetal “programming”, though beneficial for survival in a suboptimal nutritional environment, may lead to a higher risk of chronic diseases following improved nutrition and catch-up growth later in life. This hypothesis is supported by several large epidemiological studies, which show a link between the incidence of poor fetal growth, rapid catch-up growth, and susceptibility to the development of type 2 diabetes (T2DM), hypertension, cardiovascular diseases, and obesity later in life [3,4,5,6,7,8,9,10,11]. Furthermore, studies in adults and children born after IUGR indicate that insulin resistance is the earliest component associated with low birth weight, irrespective of confounding factors, including obesity and a family history of T2DM [12]. Reduced insulin sensitivity is also reported in preterm born infants during childhood, adolescence, and early adulthood [13].

Thus, an optimal early diet for infants and young children should not only support the rapid growth and development during infancy and childhood, but ideally, it should also reduce the risk of metabolic disease development later in life. The diet of early life—milk—consists of two major protein types (casein and whey), which differ not only in physiochemical properties, but also in their metabolic responses [14]. The whey-to-casein ratio of human milk varies depending on the stage of lactation (between 80:20 and 70:30 in early lactation and 50:50 in late lactation) [15].

The main differences between casein and whey with potential metabolic influences reside in their amino acid profiles, gastric emptying, satiation capacity, effect on growth hormones (insulin, IGF-1, etc.), and kinetics of amino acid delivery. For example, whey has a higher content of branched chain amino acids (BCAA), in particular, leucine, which are reported to play a role in the maintenance of lean body mass during weight loss [16,17]. However, the effect of intact protein cannot be explained solely by its amino acid profile and skim milk (a mixture of casein and whey) is reported to be more effective in preserving the lean mass of rats during weight loss than whey alone [18]. Whey and casein also exert differential effects on growth hormones by increasing either fasting insulin (whey) or circulating IGF-1 (casein) [19]. Whey also induces a greater postprandial insulin response than casein in healthy and type 2 diabetic subjects, but with no corresponding effect on blood glucose [20,21].

Whether these observed metabolic differences between whey and casein during adulthood could also occur during the rapid growth phase of early life and whether these differences have long term health consequences is not known. To address these questions, we have used a previously validated rat model of IUGR for early catch up growth and later development of impaired glucose homeostasis and excess adiposity [22] to investigate the short term and long term consequences of milk protein type (casein vs. whey) for a few weeks during the post suckling period (referred to hereafter as the weaning period, which corresponds to the complementary feeding period in the infant) on markers of glucose and insulin metabolism and body composition. The primary outcome of the study was to assess the effect of casein versus whey during the post-suckling period on long term glucose response in an IUGR model (i.e., potential programming effect).

## 2. Material and Methods

**Figure 1:**
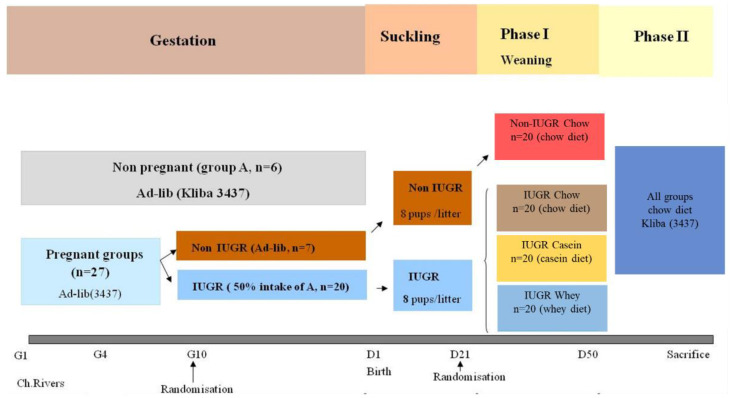
Study Design. Study flow chart including randomization and interventions during the prenatal, birth, and suckling periods. Ch. Rivers, Charles River; IUGR, intrauterine growth restriction; Ad-lib, ad-libitum.

This study was approved by the Office Vetérinaire Cantonal Vaudois (#1972). The study flow chart including randomization and interventions during the prenatal, birth, and suckling periods is illustrated in Figure 1. Briefly, the overall approach used in this study was to investigate the effect of different protein sources during weaning (casein or whey) on short- and long-term glucose homeostasis and adiposity in an IUGR model. The IUGR rats were firstly generated and a non-IUGR group was used as a control to demonstrate the phenotype (a significantly lower body weight at birth in the IUGR group due to gestational food restriction with a normal chow diet and without any dietary modification). Secondly, we compared the casein IUGR and whey IUGR animals to IUGR animals receiving the reference chow diet. Detailed materials and methods are described below.

### 2.1. Animals and Diets

Coupling conditions and Gestational period—Non-pregnant female Sprague-Dawley (SD) rats (*n* = 6) and programmed-mated SD rats (*n* = 27), aged 10–11 weeks old and with a mean body weight (± standard deviation) of 240 (±11) g (range of 233–269 g) were purchased from Charles River (France). Throughout the study, animals were housed individually in a room at 23 ± 1 °C, with 55% relative humidity and a 12 h light/dark cycle.

Female virgin SD rats were single-coupled during their estrus cycle with male SD rats of 9–11 weeks old with a mean body weight (± SD) of 371 (± 11) g (range of 324–404 g). The coupling exposure was limited to only 20 h, after which 27 animals with expelled vaginal plugs and 6 non-pregnant rats were selected for our study three days after mating. The female rats were then caged individually and fed ad libitum on a laboratory chow diet (Kliba 3437; Provimi, CH-4303 Kaiseraugst, Switzerland) until day 10 of gestation, after which the gestating rats were randomly assigned into two groups (IUGR and Non-IUGR) with a similar mean body weight. The food intake of the IUGR group (*n* = 20) was restricted to 50% of the intake of non-pregnant rats on a daily basis during the final 11 days of gestation, while the non-IUGR group (*n* = 7) continued to be fed ad-libitum until delivery (Figure 1).

Suckling period—After birth, only dams bearing at least 8 pups and with a minimum of 3–4 males per litter were selected to continue the study. The number of pups in each litter was limited to 8, with preference to male pups, and they suckled from their own mother until the age of 21 days. All dams were fed ad-libitum with a rat chow diet (Kliba 3437 Provimi, CH-4303 Kaiseraugst, Switzerland).

Phase I: (diet intervention during weaning period from the age of 21 to 49 d). After the suckling period, 60 male pups from the IUGR group were separated from dams and randomly divided into three groups (*n* = 20/group). The same number of pups from each litter was allocated to each group in order to have close maternal origin in each of the IUGR groups. In addition, 20 male pups from the Non-IUGR group were also selected as the reference group for the chow IUGR group, which had no intervention (chow IUGR). All four groups had similar mean (± standard deviation) body weight and the animals were caged individually. The Non-IUGR group (chow non-IUGR) and one of the IUGR groups (chow IUGR) were fed ad-libitum on a laboratory chow diet (kliba 3437; soybean as the main protein source), while the other two IUGR groups were fed ad-libitum on a semi-synthetic modified AIN-93G diet with either casein (casein IUGR) or whey (whey IUGR) as a protein source since it was difficult to reformulate the Kliba to change the protein composition, whereas the AIN-93G diet can be more easily modified. The diets of both casein IUGR and whey IUGR were similar (on weight basis; 53% corn starch, 10% sucrose, 7% soybean oil, 5% cellulose, 3.5% AIN-93G mineral mix, 1% AIN-93 vitamin mix, 0.25% choline bitartrate, 0.0014% tert-butylhydroquinone), except for protein source (20% casein and 0.3% L-cystine in casein IUGR and 20.3% whey in whey IUGR). The contribution of protein, fat, and carbohydrate to the energy content of both diets was 20%, 16%, and 64%, respectively. Detailed information can be found in Table 1.

Phase II (from the age of 7 to 23 weeks)—All animals were fed a laboratory chow diet (Kliba 3437). At the end of the study (age 196–199 days), different organs and tissues were collected after 6 h of daytime food deprivation (7 am–13 pm), weighed, frozen in dry ice, and kept at −80 °C until analysis.

### 2.2. Food Intake, Body Weight, and Body Composition

The food intake and body weight of dams (IUGR and non-IUGR groups) were assessed during gestation and lactation. This was performed to determine the level of food restriction required during pregnancy (more details can be found in our previous paper [22]) and to compare the food intake and body weights of all dams during pregnancy and lactation. Food intake and body weight of the dams and pups were measured 2–3 times per week throughout the study, except during the last 11 days of gestation when the food intake of dams was measured five times per week, namely during weekdays. Since food intake and body weights of dams in the IUGR groups did not differ significantly, data are not presented here. Body composition (fat mass and lean mass) of the offspring was assessed with nuclear magnetic resonance (NMR) using EchoMRI ^TM^ 2004 (Echo medical systems, Houston, TX, USA) at the age of 42–44, 120–122, 190–192 days, with a similar number of animals from each group per day.

### 2.3. Blood and Tissue Collection

Blood samples were taken from their tails after 6 h of daytime food deprivation (from 7:30 hours to 13:30 hours; referred to as fasting) at the ages of 42–44 days, 120–122 days, and 190–192 days. All blood sampling was performed with a similar number of animals from each group per sampling days. The intraperitoneal glucose tolerance test (IPGTT) was performed at the age of 190–192 days. The IP injection was performed by an experienced technician. The animal was held in the appropriate head-down position. Anatomical landmarks (e.g., hips, genitals, midline) were identified in order to inject into the appropriate area of the animal’s lower right quadrant. The needle was inserted at a 30–40° angle to the horizontal and at the correct depth to ensure it reached the abdominal cavity. Animals were monitored for some time after injection to verify the absence of diarrhea, which indicated that the injection was indeed placed in the abdomen and not in the intestine. The same procedure was followed for all animals to ensure consistency. Two blood samples were taken from the tail vein, with at least a 10-min interval between sampling (time −10 and 0), followed by an intraperitoneal injection (IP) of glucose solution (30% wt/v) at a dose of 2 g/Kg body weight. Glucose was assessed in blood samples at baseline (mean time of −10 & 0) and 15, 30, 45, 60, 90, and 120 min after glucose administration. Blood was also collected in EDTA-coated tubes for plasma insulin analyses at time 0 and 15, 30, and 60 min after IP glucose administration.

At age 196–199 days, blood samples were collected into tubes containing EDTA. Plasma was separated by centrifugation at 2200 rpm for 10 min. Plasma samples were kept frozen in dry ice and kept at −80 °C until analysis. Different organs (heart, liver, kidney, gastrocnemius, and spleen) and adipose tissue (epididymal and retroperitoneal) were separated and weighed.

### 2.4. Blood Assays

Blood glucose was measured using Ascensia Elite XL glucometers (Bayer AG, Zurich, Switzerland). Plasma insulin and leptin were measured by the ELISA method using appropriate kits from Crystal Chem. Inc (Elk Grove Village, IL, USA), Linco (now Millipore) (Burlington, MA, USA). Plasma triacylglycerol (TG), cholesterol (Chol), and glucose were measured by enzymatic methods with a centrifugal analyzer (Cobas FARA, Roche Diagnostica, Basel, Switzerland) using appropriate kits and calibration solutions from BioMérieux (Marcy-l’Etoile, France). Plasma free fatty acids were analyzed by Cobas FARA (Roche Diagnostica, Basel, Switzerland) using a NEFA C kit (Wako, Neuss, Germany).

### 2.5. Statistical Analysis

**Figure 2:**
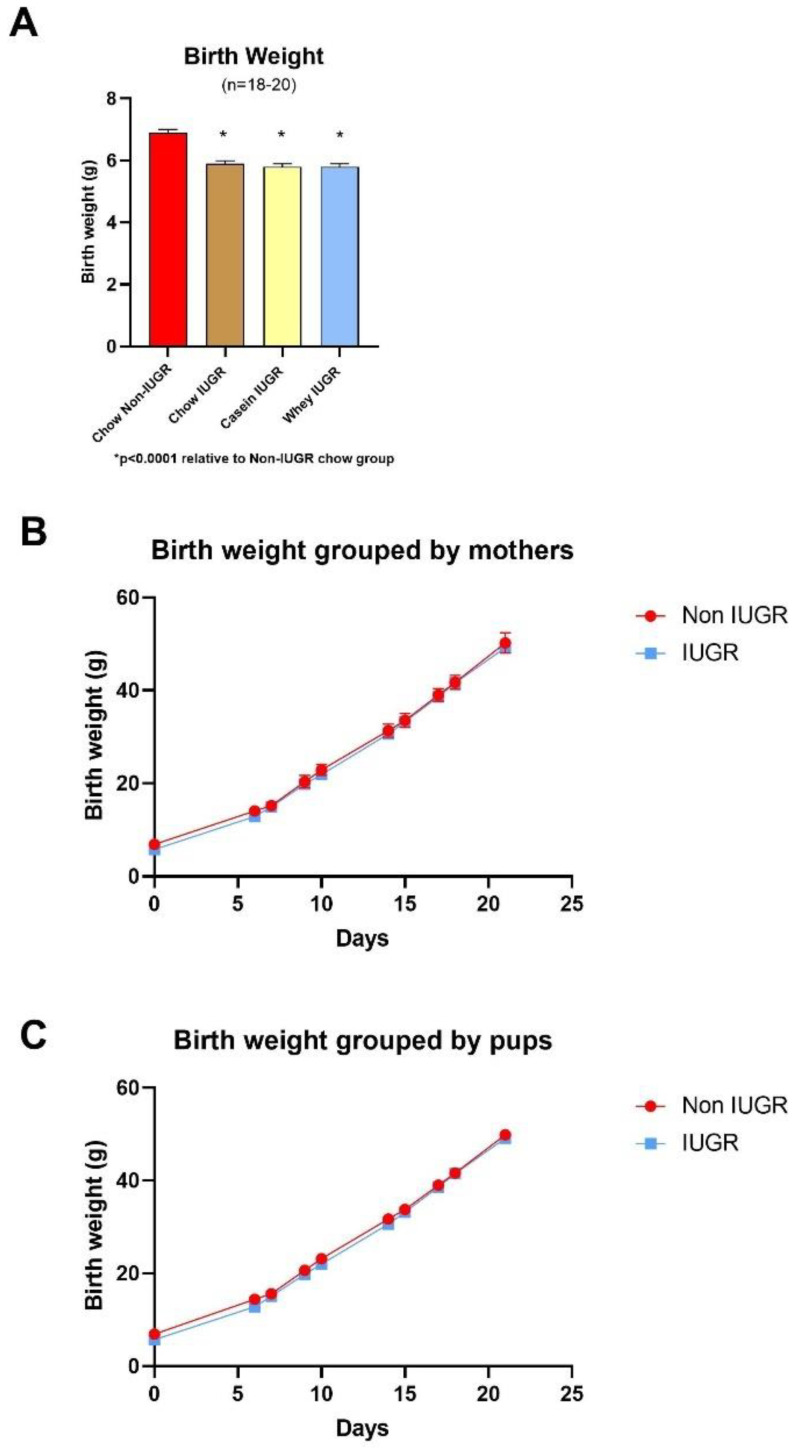
Birth weight. (**A**) Birth weight in grams; (*n* = 18–20); (**B**) birth weight grouped by litter (IUGR *n* = 14; non-IUGR *n* = 6) and (**C**) birth weight grouped by pups (IUGR *n* = 58; non-IUGR *n* = 19). All IUGR groups were significantly lower than non-IUGR. Values are mean ± SEM.

Due to the data distribution, body weight parameters and food intake were analyzed using classical parametric statistics, while for all the other parameters, non-parametric statistics (median, robust SD, Kruskal Wallis test) were used. Groups were compared as follows: (i) non-IUGR chow vs. IUGR chow, (ii) chow IUGR vs. casein IUGR, (iii) chow IUGR vs. whey IUGR, and (iv) casein IUGR vs. whey IUGR. The only exception to this is the data before weaning (i.e., during the suckling period; Figure 2). Since the casein and whey diets were only implemented in the post-suckling/weaning period, for the suckling period, we only compared non-IUGR with IUGR-chow. For IPGTT, the total Area under the Curve (AUC) for glucose (120 min) and for insulin (60 min) was calculated using the trapezoidal rule. These variables are presented in the text as Median ± SEM. Food intake data were analyzed by ANOVA and appropriate contrasts and results are presented as Mean ± SEM. The analysis of BW and BWG were performed by two overlapping (around day 50) mixed models with group and time (day) as continuous variables, their interaction as fixed effects, and subject as a random variable (intercept and slope). These variables are presented as Mean ± SEM. The time of catch up growth was calculated using bootstrap to assess the mean and the 95% confidence level. As the primary objective corresponds to one comparison and a hierarchy was set in the secondary outcomes, no correction for multiple testing was applied. All tests were therefore performed using a significance level of alpha = 5%. The statistical analysis was performed using the software SAS 9.1.

## 3. Results

### 3.1. Birth Weight and Time of Catch-Up Growth

The gestational food restriction resulted in a significant reduction in birth weight of the IUGR pups relative to the non-IUGR group, whether per litter analysis (median weight of pups in each litter) or individually (per pup); the mean birth weight reductions were −1.06 g for the pups (range: −0.86 to −1.26 g) (*p* < 0.0001). As shown in Figure 2A, the birth weight of pups in all IUGR groups was significantly lower than that of the non-IUGR groups (−13% in the Chow IUGR group and −15% in both the Casein IUGR and Whey IUGR groups (*p* < 0.0001, in all cases)). During the suckling (i.e., lactation) period, the pups in the IUGR groups showed catch-up growth. Table 2 shows the time taken for completing the catch-up growth, calculated based on litter (median weight of pups/litter) or pups individually as sampling unit and for each treatment group, even if treatment was not yet started.

The catch-up growth occurs during the suckling period and before the start of any treatment. The time of catch-up growth was similar in IUGR pups allocated to the different groups and occurred at the age of 9–11 days on a per litter unit and 11–13 days on a per pup unit. However, as indicated by the 95% CI range, there was a large variation of over 15 days with both sampling units used (per litter or per pup).

### 3.2. Weaning and Post-Weaning Growth, Body Composition, and Energy Intake

**Figure 3:**
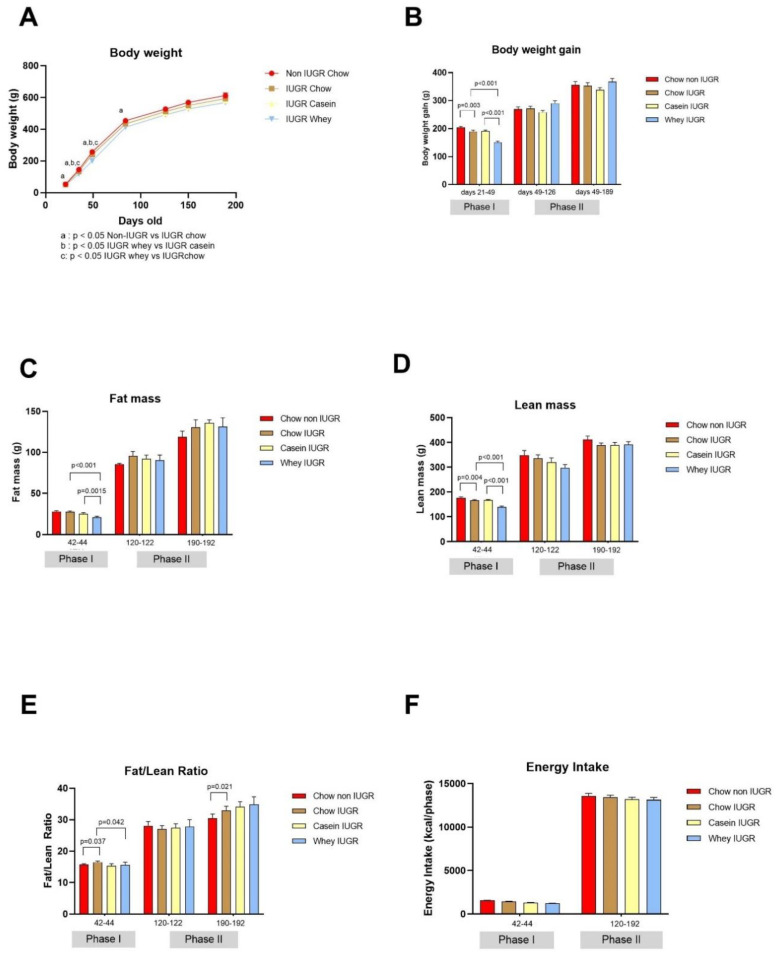
Body weight, body composition, and energy intake. (**A**) Body weight in grams of each group (*n* = 19 chow non-IUGR; *n* = 20 chow IUGR; *n* = 20 casein IUGR; *n* = 19 whey IUGR); values are mean ± SEM; (**B**) Body weight gain in grams per phase of the study (*n* = 19–20 all Phases), values are mean ± SEM; (**C**) Fat mass in grams for each group (*n* = 19 chow non-IUGR; *n* = 20 chow IUGR; *n* = 20 casein IUGR; *n* = 19 whey IUGR), values are median ± SE median; (**D**) Lean mass in grams for each group (*n* = 19 chow non-IUGR; *n* = 20 chow IUGR; *n* = 20 casein IUGR; *n* = 19 whey IUGR), values are median ± SE median; (**E**) Fat/lean ratio for each group (*n* = 19 chow non-IUGR; *n* = 20 chow IUGR; *n* = 20 casein IUGR; *n* = 19 whey IUGR); (**F**) Energy intake (kcal/phase) *n* = 19–20 in each phase, values are mean ± SEM.

Following catch-up growth during the suckling period, non-IUGR and IUGR pups had a similar median body weight when expressed per litter (Figure 2B) and per pups (Figure 2C), as well as in all IUGR groups (age 20 days, Figure 3A). However, after separation from the dams and upon weaning to the different diets during phase I (age 21–49), the IUGR groups showed a slower rate of growth than the non-IUGR group (Figure 3A). This was associated with the lower weight gain during this period (Figure 3B), particularly in the whey IUGR group (Figure 3B). At the age of 42–44 days, the mean body weight, body fat, and lean mass of the whey IUGR group were significantly lower than that of the casein IUGR group by 21%, 16.7%, and 15.4%, respectively (all at *p* < 0.001), but not the fat-to-lean mass ratio (median ± SE median; 15.6 ± 0.9 and 15.3 ± 0.7, respectively) (Figure 3B–E). Although during this period, the energy intake of the whey IUGR group was also 9% lower than that of casein IUGR, this difference was not significant (*p* < 0.07) (Figure 3F). During Phase I, the body weight gain (*p* < 0.001), fat mass (*p* < 0.001), lean mass (*p* < 0.001), and fat-to-lean mass ratio (*p* = 0.042) of the IUGR whey group were significantly lower than the IUGR chow group. During phase I, chow IUGR vs. chow non-IUGR also had lower weight gain (*p* < 0.001) that was associated with lower energy intake (*p* = 0.03) and was reflected in a lower lean mass (*p* < 0.001), but not fat mass (*p* > 0.05) (Figure 3B–D). The fat-to-lean mass ratio was significantly higher in the chow IUGR group (median ± SE median; 16.5 ± 0.4 and 15.7 ± 0.3, *p* < 0.04) (Figure 3E).

During phase II (age 49–190 days), when all groups were fed on the chow diet, they showed no differences in energy intake (Figure 3F). The body weight was lower in IUGR groups, particularly in the whey IUGR group (Figure 3A). At the age of 190–192 days, body weight remained lower in the IUGR groups than in the non-IUGR group and also lower in the group weaned on the whey diet than in those weaned on the casein diet, but these differences in body weight and body weight gain (Figure 3B) were not statistically significant. Similarly, analysis of body composition revealed a lower lean mass and greater fat and fat-to-lean mass ratio in the IUGR groups than in the non-IUGR group, but these differences were only statistically significant for fat-to-lean mass ratio between chow groups (32.9 ± 1.4 vs. 30.5 ± 1.3, *p* < 0.03, respectively) (Figure 3C–E). With respect to the organs and tissues assessed, chow-IUGR group had lower kidney weight and epidydimal adipose tissue. The only significant difference between IUGR casein and IUGR whey was lower pancreas weight in the whey group (Table 3). However, comparison of the IUGR groups weaned on the casein and whey diets showed no differences in body composition, whether in their total body fat, lean mass, and fat-to-lean mass ratio (Figure 3C–E). Prenatal food restriction (non-IUGR chow vs. IUGR-chow) significantly decreased the weight of the kidney (*p* = 0.009). The protein source of early diet in IUGR rats (IUGR casein vs. IUGR whey) had a significant effect only on the weight of the pancreas, which was higher in the IUGR casein group vs. IUGR chow and IUGR whey (*p* = 0.017 and *p* = 0.036, respectively) (Table 3).

### 3.3. Blood Glucose and Insulin

**Figure 4:**
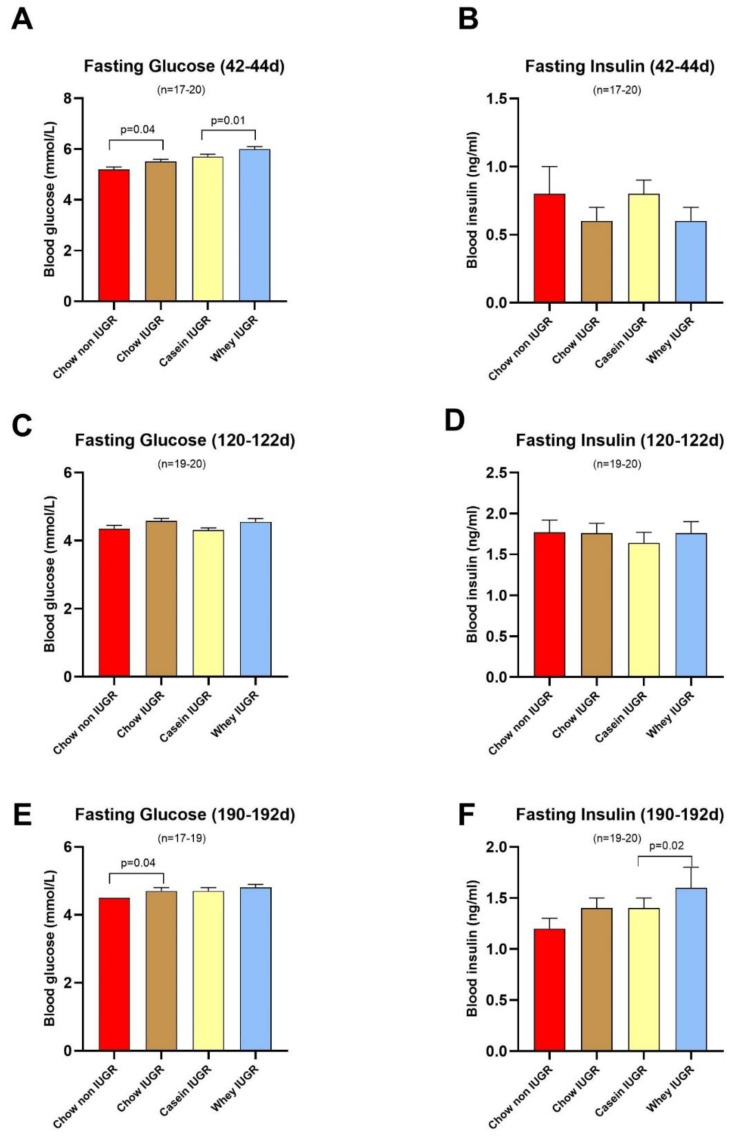
Fasting Glucose and Insulin values. Fasting glucose and insulin at days 42 ((**A**) and (**B**), respectively), 92 ((**C**) and (**D**), respectively), and 121 ((**E**) and (**F**), respectively); *n* = 17–20 per group; values are median ± SE median.

The results for fasting blood glucose and insulin are shown in Figure 4. At the end of the third week of the diet intervention (phase I, age 42–44 d), fasting plasma glucose was higher in the IUGR groups than in the non-IUGR group (Figure 4A; *p* = 0.04 between chow groups) and furthermore, it was higher in the IUGR group that was fed the whey diet compared to the casein diet (Figure 4A; *p* = 0.01). No significant differences were observed among the groups for fasting plasma insulin (Figure 4B).

Similarly, at the age of 121 days during phase II, when all groups were fed the chow diet, there were no differences in fasting plasma insulin, but a tendency for fasting plasma glucose to be higher in the IUGR groups than in the non-IUGR group, with the value being higher in IUGR group weaned on the whey diet than on the casein diet (Figure 4C; *p* = 0.06). There was no difference of fasting insulin in any groups (Figure 4D). At age 190–192 days (phase II), there was also a higher fasting plasma glucose in the IUGR groups than the non-IUGR group (Figure 4E; *p* = 0.04 between chow groups). Although the change in fasting plasma glucose between the two IUGR groups weaned on the casein and whey diets did not reach statistical significance, the fasting plasma insulin was significantly higher in the IUGR group weaned on the whey diet than on the casein diet (+14%, *p* = 0.02; Figure 4F).

**Figure 5:**
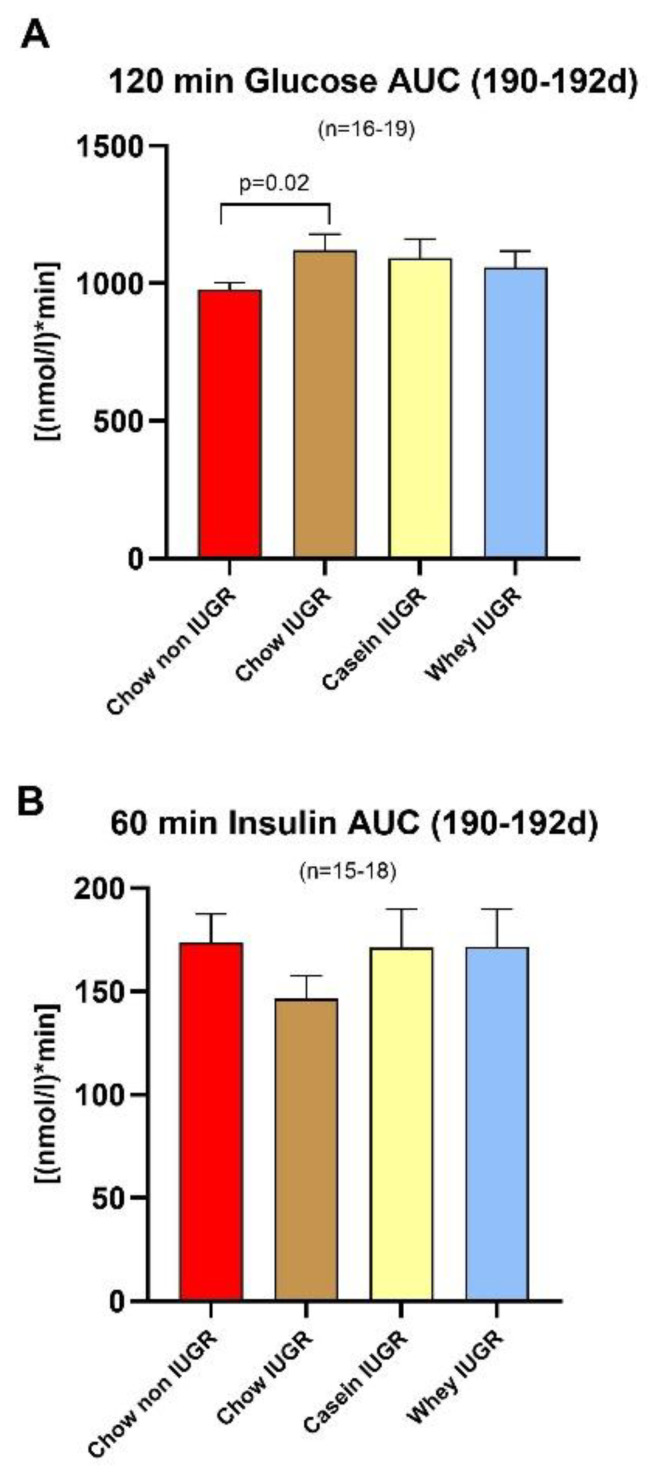
Glucose and Insulin Area under the Curve (AUC). (**A**) 120 min glucose AUC 190-192d and (**B**) 60 min Insulin AUC 190-192d; *n* = 16–19 per group for glucose and *n* = 15–18; values are median ± SE median.

A test of glucose tolerance (IPGTT) at this time point (age 190–192 days) revealed a significantly higher blood glucose response curve in the IUGR groups than in the non-IUGR group (*p* = 0.02, between chow groups), with no differences in their insulin response curves (Figure 5). Comparison between the two IUGR groups weaned on the casein and whey diets showed no significant differences in their blood glucose or insulin responses during IPGTT.

### 3.4. Evolution of the HOMA-IR Index

**Figure 6:**
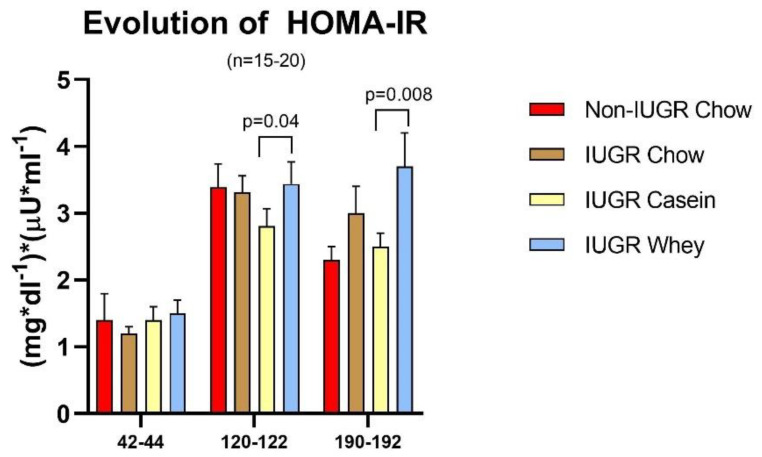
Evolution of homeostatic model assessment of insulin resistance (HOMA-IR) with age (*n* = 15–20 per group).

The homeostatic model assessment of insulin resistance (HOMA-IR), calculated from fasting plasma glucose and insulin level, was assessed as an index of insulin sensitivity at the age of 42–44, 120–122, and 190–192 days (Figure 6). During the last week of the intervention period (phase I: age 42–45 days), the HOMA-IR was not significantly different among the groups. However, at the older ages of 120–122 days and 190–192 days, the IUGR group weaned on the whey diet had higher HOMA-IR (lower insulin sensitivity) relative to the IUGR group weaned on the casein diet (+22% (*p* = 0.04) and + 48% (*p* = 0.008), respectively).

### 3.5. Plasma Lipids and Leptin

Plasma lipids and leptin were determined in samples collected at the end of the study and the data are presented in Table 4. Although there is a tendency for fasting plasma lipids (total cholesterol, TG, FFA), as well as plasma leptin, to be higher in the IUGR groups than in the non-IUGR group, these differences are not statistically different. Furthermore, there are no differences between the two IUGR groups weaned during phase I on the casein diet vs. the whey diet in any of these blood parameters.

## 4. Discussion

The aim of this study was to compare the impact of different milk protein types (casein and whey) during the post suckling period (referred to as the weaning diet in this study) on markers of glucose homeostasis and adiposity in the IUGR rat model that is sensitive for later insulin resistance and excess adiposity. We demonstrated the advantage of casein relative to whey during the weaning period for growth, body composition, and blood glucose homeostasis both in the short term and in the long term.

In the present study, the IUGR versus non-IUGR pups were born with 15% lower birth weight and subsequently underwent rapid catch-up growth during the suckling period, with completion of catch-up growth by a mean age of 13 d (confidence limit of 9 to 20 days). However, during the post-suckling period, the IUGR rats showed lower food intake and hence, lower growth velocity, resulting in a lower body weight until the age of 84 days. At the older age of 190–192 days, the IUGR rats showed lower lean mass and a higher fat-to-lean ratio, which suggests a tendency towards an obesity phenotype in the long term. Moreover, the IUGR rats also had higher fasting blood glucose in the short term (age of 42–44 days) and higher fasting glucose and glucose AUC during IPGTT in the long term (age of 190–192 days). These differential effects on glycemia in IUGR and non-IUGR groups were observed without significant effects on baseline insulin and insulin response to glucose (AUC), thereby suggesting greater insulin resistance in the IUGR group. Thus, the results of this study, in line with other reports [23,24], also confirm that our prenatal food restricted IUGR model [22] shows the development of phenotypes of excess adiposity, higher glycaemia, and glucose intolerance in adulthood. Hence, it is a suitable model for investigating the effect of early diet on later development of adiposity and glucose homeostasis in rats.

Using this validated IUGR rat model, our findings suggest that the protein source of the weaning diet has an impact on food intake, body weight, body composition, and glucose homeostasis in the short term and the development of insulin resistance in the long term. In the current study, catch-up growth occurred during the suckling period and thus, before the introduction of casein or whey to the diet. During the post-suckling period (weaning) and after catch-up growth when the casein and whey were introduced, we observed a beneficial effect of casein relative to whey. The whey IUGR group showed a tendency for lower energy intake during the weaning period (phase I), with a 22% lower weight gain at the end of the weaning period (age 49 days) relative to the casein IUGR group. However, this was reversed during Phase II, with higher weight gain in the whey IUGR group from the age of 56 days; consequently, at the end of the study, there were no significant differences in body weight between the groups. Similar findings were observed for the difference between IUGR whey vs. IUGR chow during Phase I, which were not apparent during Phase II. Considering that the growth rate of all IUGR groups was slower than that of non-IUGR during the weaning period and that the body weight of the whey group was lower than that of the casein and IUGR chow, this suggests the importance of growth during the weaning period and highlights the advantage of casein relative to whey for low-birth weight children during the weaning period. Whether this is due to the specific metabolic effects of whey protein per se and/or due to the effect of whey on growth through appetite regulation and food intake remains to be investigated. We observed differences in tissue weight between the non-IUGR and IUGR groups, which is expected and due to the prenatal food restriction. The findings related to casein and tissue weight are intriguing. However, future studies are necessary, particularly histological and functional studies of organs and tissues to explore the impact of early life protein source in order to elucidate the mechanism that might explain our observed difference between whey and casein.

To our knowledge, the impact of different types of milk protein (casein and whey) on energy intake (appetite control) and growth has not been studied in the IUGR model. However, in non-IUGR human or animal studies, the higher satiety/satiation effect of whey relative to whole milk (80% casein protein) or casein has previously been reported in some, but not all studies [25,26,27]. The mechanism of action is proposed to be via increased secretion of cholecystokinin (CCK) in response to the high level of whey glycomacropeptide (GMP) [28,29], as well as higher glucagon-like peptide-1 secretion (GLP-1), both of which are known to be involved in the induction of satiation and/or satiety [30,31]. The appetite reducing effect of whey may also be due to its greater content of BCAA (particularly leucine), which have previously been reported to influence energy intake and glucose homeostasis [16,17].

Interestingly, after three weeks of the weaning intervention (phase I: age 42–44 days), the whey IUGR group had higher fasting glycaemia, with a trend for increased glucose at 120 days. Furthermore, the fasting insulin at the age of 190–192 days and HOMA-IR (at both ages of 120–122 days and 190–192 days) was significantly higher in the IUGR rats who were previously fed with a whey diet during the weaning period relative to those fed with a casein weaning diet. These effects, which were observed 140 days after the end of the weaning intervention and during the time when all groups were fed with the same chow diet, demonstrate, for the first time, the programming potential of the protein source of the weaning diet for later development of insulin resistance in IUGR rats with an apparent advantage of casein relative to whey.

It is known that maternal physiological factors have the potential to impact offspring metabolic health including maternal age [32], maternal diet, maternal morbidity (e.g., obesity, diabetes, preeclampsia) [33,34,35,36], exercise [37,38], and maternal stress [39]. In our study, we minimized the impact of certain factors by ordering rats from Charles River with a similar age and weight. The rats were virgin and were only mated for one day; from these, the pregnant animals were then selected. In addition, they were exposed to similar environmental conditions and received the same diets since birth. Moreover, by allocating the same number of pups from each dam to each group, we tried to minimize the impact of the dams on the outcomes of the study. Lastly, we did not observe differences in maternal weight or food intake between the IUGR groups.

It has been shown that the male placenta is more susceptible to damage by adverse nutrition in-utero [40]. However, there are known sex differences in predisposition to metabolic diseases and in addition to the aforementioned effects in males, females appear to be susceptible to developing increased adiposity and altered glucose homeostasis in response to undernutrition in-utero [40]. Future studies including both sexes are needed to expand our understanding of how early life nutrition may differentially impact male and female health in later life.

## 5. Conclusions

The results of this study highlight the influence of milk protein type during the post-suckling period (referred to as the weaning period in this study) on energy intake, growth, and glucose homeostasis in the short term and reveal, for the first time, its long term impact on insulin sensitivity later in life. These findings suggest a potential advantage of casein relative to whey in IUGR animals during the weaning period. This may have implications for later metabolic health, particularly in small-for-gestational age populations at risk of T2DM.

## Figures and Tables

**Table 1:** Composition of the study diets.

Diet Component	Kliba 3437	Casein Diet	Whey Diet
Gross energy (kcal/100 g)	382	365	365
Protein (% energy)	19.4	20	20
Fat (% energy)	11	16	16
Carbohydrate (% energy)	57	64	64
Fiber (% energy)	4.7	4.8	4.8
Amino acids (%protein)			
Aspartame	-	7.5	11.5
Threonine	3.5	4.8	5.1
Serine	-	4.8	5.1
Glutamine	-	22.8	18.1
Proline	-	10.9	5.2
Glycine		2.1	2.0
Alanine	-	3.2	5.1
Valine	-	6.8	5.7
Cysteine	-	0.4	2.7
Methionine	2.2	2.8	2.3
Isoleucine	-	5.8	5.7
Leucine	-	10.4	13.1
Tyrosine	-	5.9	4.0
Phenylalanine	-	5.0	3.8
Lysine	5.4	8.4	10.3
Histidine	-	2.9	2.2
Arginine	5.6	4.0	2.8
Tryptophan	1.2	1.4	2.2

Dashed lines indicate values not provided by the producer. Protein composition of the diets was provided by the producer.

**Table 2:** Time of catch up growth (day of suckling period) in litters and pups.

		Bootstrap Mean	Approximate	Approximate Lower	Approximate Upper
		Age (Day)	Standard Error	Confidence Limit	Confidence Limit
Litter	IUGR Chow	9.1	3.06	6.4	18.4
	IUGR Casein	11.1	3.32	7.4	20.4
	IUGR Whey	10.5	3.22	6.5	19.2
Pup	IUGR Chow	10.6	3.29	7.2	20.1
	IUGR Casein	13.4	2.76	9.1	19.9
	IUGR Whey	12.7	2.76	8.7	19.5
Litter IUGR		11.2	3.31	7.2	20.2
Pups IUGR		12.8	2.84	8.6	19.7

**Table 3:** Tissue/organ weight at the end of the study (197–199 days).

	Epidydimal	Retroperitoneal	Kidney	Liver	Pancreas
Non IUGR Chow	9.72 (0.73)	7.35(0.8)	3.34 (0.1) ^a^	17.7 (0.84)	1.22 (0.04)
IUGR Chow	10.9 (1.25)	9.44 (0.74)	3.2 (0.09) ^b^	18.02 (0.48)	1.17 (0.06) ^a^
IUGR Casein	9.55 (0.72)	8.86 (0.58)	3.22 (0.09)	16.53 (0.42)	1.34 (0.05) ^b^
IUGR Whey	10.07 (0.79)	10.56 (1.24)	3.08 (0.07)	17.21 (0.5)	1.17 (0.06) ^a^

Values are median (SE median) weight in grams; *n* = 18–20/group; different letters indicate significant within-column differences (*p* < 0.05). IUGR, intrauterine growth restriction. Groups were compared as follows: (i) Non-IUGR chow vs. IUGR chow; (ii) IUGR chow vs. IUGR casein; (iii) IUGR chow vs. IUGR whey; (iv) IUGR casein vs. IUGR whey. Only significant comparisons are highlighted.

**Table 4:** Blood glucose, plasma insulin, fatty acids, and cholesterol at the end of the study (197–199 days).

	Leptin	FFA	TG	Total Cholesterol
	ng/mL	µmol/L	mmol/L	mmol/L
Non IUGR Chow	5.75	360.5 (32.4)	0.99 (0.11)	2.47 (0.12)
IUGR Chow	5.87	363 (19.0)	1.13 (0.12)	2.7 (0.13)
IUGR Casein	7.08	419.75 (33.1)	0.96 (0.14)	2.54 (0.09)
IUGR Whey	6.85	419 (26.4)	1.04 (0.12)	2.8 (0.18)

Values are median (SE median); *n* = 18–20/group.

## Abbreviations

AUC: area under the curve; BCAA, branched chain amino acids; BW, body weight; BWG, body weight gain; CCK, cholecystokinin; FFA, free-fatty acids; GIP, glucose-dependent insulinotropic polypeptide; GLP-1, glucagon-like peptide-1; HOMA IR, homeostatic model assessment of insulin resistance; IGF-1; insulin-like growth factor-1; IP, intraperitoneal injection; IPGTT, intraperitoneal glucose tolerance test; IUGR, intrauterine growth restriction; NEFA, non-esterified fatty acids; NMR, nuclear magnetic resonance; SD rats, Sprague-Dawley rats; TG, triglyceride.
